# Supplementary figures and images for: Lack of impact of OCTN1 gene polymorphisms on clinical outcomes of gabapentinoids in Pakistani patients with neuropathic pain
Source: PLoS One. 2022 May 13;17(5):e0266559. doi: 10.1371/journal.pone.0266559 (PMC9106170; doi:10.1371/journal.pone.0266559)

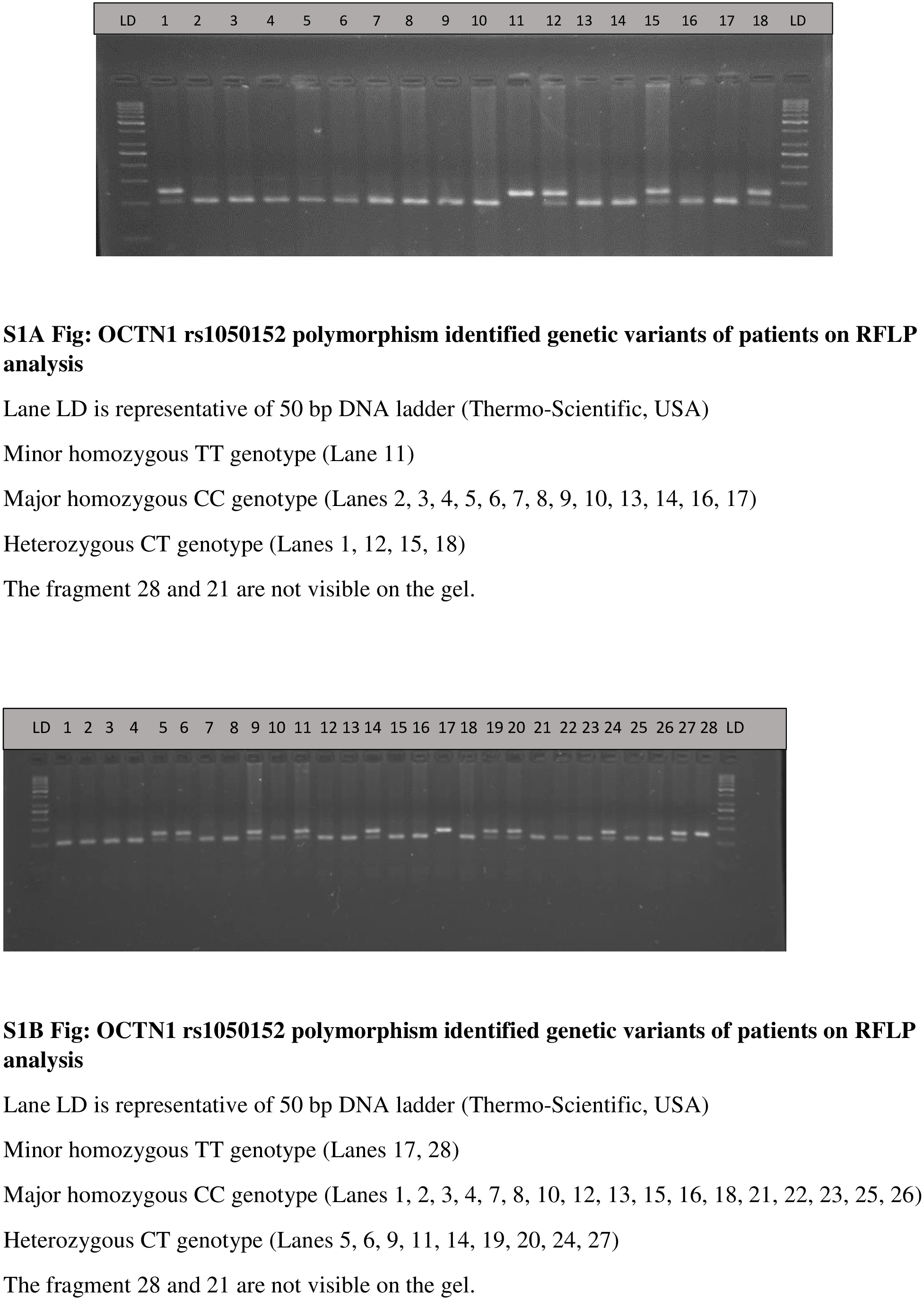

Supplement: S1 Fig — A. OCTN1 rs1050152 polymorphism identified genetic variants of patients on RFLP analysis. B. OCTN1 rs1050152 polymorphism identified genetic variants of patients on RFLP analysis. (TIF) [file pone.0266559.s001.tif]

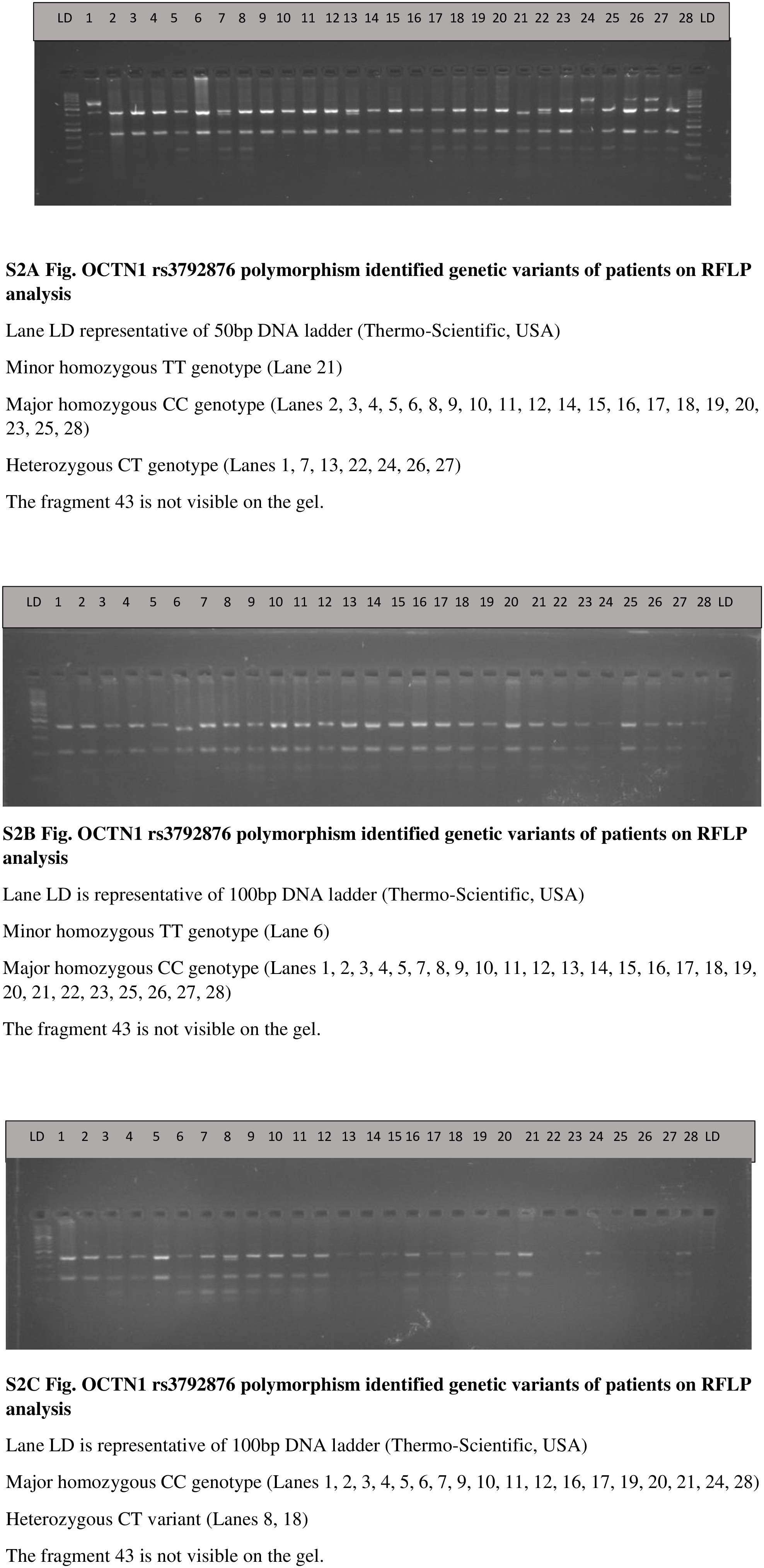

Supplement: S2 Fig — A. OCTN1 rs3792876 polymorphism identified genetic variants of patients on RFLP analysis. B. OCTN1 rs3792876 polymorphism identified genetic variants of patients on RFLP analysis. C. OCTN1 rs3792876 polymorphism identified genetic variants of patients on RFLP analysis. (TIF) [file pone.0266559.s002.tif]
