# Supplementary material for: Lack of impact of OCTN1 gene polymorphisms on clinical outcomes of gabapentinoids in Pakistani patients with neuropathic pain
Source: PLoS One. 2022 May 13;17(5):e0266559. doi: 10.1371/journal.pone.0266559 (PMC9106170; doi:10.1371/journal.pone.0266559)
Supplement: S1 Table — (DOCX) [file pone.0266559.s003.docx]

**S1 Table: Comparison of baseline demographics and clinical characteristics among different genotypes of OCTN1 rs1050152**

|  | **TT (n=14)** | **CT (n= 108)** | **CC (n=279)** | ***P*-value** |
| --- | --- | --- | --- | --- |
| **Gender n(%)**  Female  Male | 10 (2.49)  4 (0.997) | 60 (14.96)  48 (11.97) | 150 (37.40)  129 (32.17) | 0.449 |
| **Age (y)** (Mean ± SD) | 50.64 ± 7.69 | 51.95 ± 9.29 | 51.78 ± 10.12 | 0.895 |
| **Weight (kg)** (Mean ± SD) | 72.80 ± 4.76 | 72.74 ± 5.67 | 72.99 ± 6.42 | 0.933 |
| **Ethnicity** n (%)  Kashmiri  Urdu Speaking  Pathan  Punjabi  Others | 0 (0)  1 (0.25)  5 (1.25)  8 (1.995)  0 (0) | 3 (0.75)  4 (0.997)  11 (2.74)  87 (21.70)  3 (0.7%) | 12 (2.99)  18 (4.49)  37 (9.23)  202 (50.37)  10 (2.49) | 0.245 |
| **Serum creatinine** **(mg/dl)** (Mean ± SD) | 0.90 ± 0.20 | 0.83 ± 0.16 | 0.86 ± 0.16 | 0.152 |
| **eGFR (ml/min/1.73m^2^)** (Mean ± SD) | 82.71 ± 26.18 | 91.25 ± 19.32 | 88.73 ± 21.10 | 0.281 |
| **Etiology** n (%)  CPRS  Intercostal neuralgia  Radicular pain  Painful Diabetic Neuropathy  Others | 1 (0.25)  0 (0)  3 (0.75)  6 (1.50)  4 (0.997) | 3 (0.75)  3 (0.75)  34 (8.48)  55 (13.72)  13 (3.24) | 6 (1.50)  10 (2.49)  67 (16.71)  149 (37.16)  47 (11.72) | 0.547 |
| **Baseline pain score**  (Mean ± SD) | 7.0 ± 0.55 | 6.95 ± 0.68 | 6.92 ± 0.68 | 0.871 |
| **Pregabalin dose (mg/day)**  (Mean ± SD) | 110.00 ± 74.72  (10) | 104.10 ± 53.58  (67) | 109.16 ± 52.95  (191) | 0.798 |
| **Gabapentin dose (mg/day)**  (Mean ± SD) | 450.00 ± 173.205  (4) | 514.63 ± 200.70  (41) | 521.02 ± 213.04  (88) | 0.798 |
