# Supplementary material for: Lack of impact of OCTN1 gene polymorphisms on clinical outcomes of gabapentinoids in Pakistani patients with neuropathic pain
Source: PLoS One. 2022 May 13;17(5):e0266559. doi: 10.1371/journal.pone.0266559 (PMC9106170; doi:10.1371/journal.pone.0266559)
Supplement: S2 Table — (DOCX) [file pone.0266559.s004.docx]

**S2 Table: Comparison of baseline demographics and clinical characteristics among different genotypes of OCTN1 rs3792876**

|  | **TT (n=16)** | **CT (n=60)** | **CC (n=322)** | ***P*-value** |
| --- | --- | --- | --- | --- |
| **Gender** n (%)  Male  Female | 5 (1.26)  11 (2.76) | 26 (6.53)  34 (8.54) | 150 (37.69)  172 (43.22) | 0.455 |
| **Age (y)** (Mean ± SD) | 54.06±9.62 | 51.40 ± 9.72 | 51.71±10.09 | 0.628 |
| **Weight (kg)** (Mean ± SD) | 71.63±5.46 | 72.72±6.09 | 73.09±6.28 | 0.615 |
| **Ethnicity** n (%)  Kashmiri  Urdu Speaking  Pathan  Punjabi  Others | 0 (0)  1 (0.25)  2 (0.51)  13 (3.27)  0 (0) | 2 (0.51)  2 (0.51)  13 (3.27)  42 (10.55)  1 (0.25) | 11 (2.76)  17 (4.27)  41 (10.30)  245 (61.56)  8 (2.01) | 0.781 |
| **Serum creatinine** **(mg/dl)** (Mean ± SD) | 0.83 ± 0.11 | 0.84 ± 0.16 | 0.86 ± 0.16 | 0.675 |
| **eGFR (ml/min/1.73m^2^)** (Mean ± SD) | 86.44 ± 19.44 | 90.75 ± 20.47 | 89.68 ± 20.72 | 0.758 |
| **Etiology** n (%)  CPRS  Intercostal neuralgia  Radicular pain  Diabetic Peripheral Neuropathy  Others | 0 (0)  0 (0)  3 (0.77)  12 (3.02)  1(0.25) | 0 (0)  0 (0)  11 (2.76)  37 (9.30)  12 (3.02) | 10 (2.51)  14 (3.52)  92 (23.12)  155 (38.94)  51 (12.81) | 0.107 |
| **Baseline pain score**  (Mean ± SD) | 6.88±0.62 | 7.00±0.69 | 6.90±0.67 | 0.575 |
| **Pregabalin dose (mg/day)**  (Mean ± SD) | 112.50 ±51.68  (12) | 105.68 ±55.21  (44) | 108.57±54.49  (207) | 0.915 |
| **Gabapentin dose (mg/day)**  (Mean ± SD) | 562.50± 256.174  (4) | 500±202.49  (16) | 514.78 ±207  (115) | 0.864 |

**S3 Table: Candidate genes SNP markers, primer sequences, product size, restriction enzymes, annealing temperatures**

| **SNPs** | **Primer sequences 5’-3’** | **Product size (bp)** | **PCR AT**  **(°c)** | **REs** | **Fragment size (bp)** |
| --- | --- | --- | --- | --- | --- |
| **OCTN1 (rs1050152)** | **F-5’-TCCCAAACTTTCAGGGAAAA-3’**  **R-5’-CAAGAGTGCCCAGAGAGTCC-3’** | **151** | **60°c** | **MnlI** | CC 102, 28, 21  CT 123, 102, 28, 21  TT 123, 28 |
| **OCTN1 (rs3792876)** | **F-5’-AGGCTAAAGGAGCAGGAAG-3’**  **R-5’-TCTCAGTGCCTCCCAGAAGT-3’** | **620** | **60°c** | **HphI** | CC 412, 208  CT 412, 369, 208, 43  TT 369, 208, 43 |

SNPs: Single nucleotide polymorphisms; OCTN1: Organic Cation Transporter1; bp; base pairs; PCR: Polymerase chain reaction; AT: Annealing temperature
