# Supplementary material for: Lack of impact of OCTN1 gene polymorphisms on clinical outcomes of gabapentinoids in Pakistani patients with neuropathic pain
Source: PLoS One. 2022 May 13;17(5):e0266559. doi: 10.1371/journal.pone.0266559 (PMC9106170; doi:10.1371/journal.pone.0266559)
Supplement: S1 Raw images — (PDF) [file pone.0266559.s005.pdf]

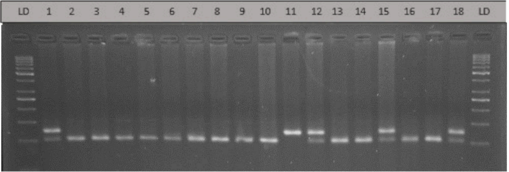

S1A Fig: OCTN1 rs1050152 polymorphism identified genetic variants of patients on RFLP analysis

Lane LD is representative of 50 bp DNA ladder (Thermo-Scientific, USA); Minor homozygous TT genotype (Lane 11); Major homozygous CC genotype (Lanes 2, 3, 4, 5, 6, 7, 8, 9, 10, 13, 14, 16, 17); Heterozygous CT genotype (Lanes 1, 12, 15, 18); The fragment 28 and 21 are not visible on the gel.

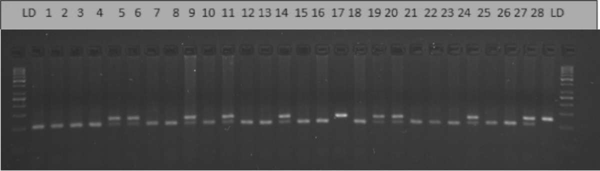

S1B Fig: OCTN1 rs1050152 polymorphism identified genetic variants of patients on RFLP analysis

Lane LD is representative of 50 bp DNA ladder (Thermo-Scientific, USA); Minor homozygous TT genotype (Lanes 17, 28); Major homozygous CC genotype (Lanes 1, 2, 3, 4, 7, 8, 10, 12, 13, 15, 16, 18, 21, 22, 23, 25, 26); Heterozygous CT genotype (Lanes 5, 6, 9, 11, 14, 19, 20, 24, 27); The fragment 28 and 21 are not visible on the gel.

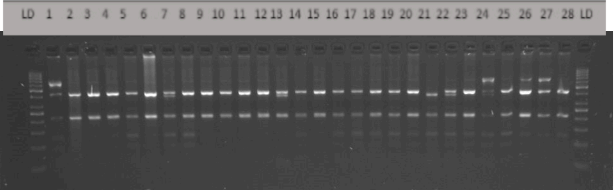

S2A Fig. OCTN1 rs3792876 polymorphism identified genetic variants of patients on RFLP analysis

Lane LD representative of 50bp DNA ladder (Thermo-Scientific, USA); Minor homozygous TT genotype (Lane 21); Major homozygous CC genotype (Lanes 2, 3, 4, 5, 6, 8, 9, 10, 11, 12, 14, 15, 16, 17, 18, 19, 20, 23, 25, 28); Heterozygous CT genotype (Lanes 1, 7, 13, 22, 24, 26, 27); The fragment 43 is not visible on the gel.

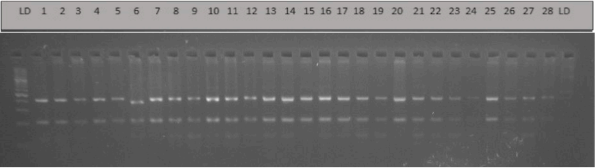

S2B Fig. OCTN1 rs3792876 polymorphism identified genetic variants of patients on RFLP analysis

Lane LD is representative of 100bp DNA ladder (Thermo-Scientific, USA); Minor homozygous TT genotype (Lane 6); Major homozygous CC genotype (Lanes 1, 2, 3, 4, 5, 7, 8, 9, 10, 11, 12, 13, 14, 15, 16, 17, 18, 19, 20, 21, 22, 23, 25, 26, 27, 28); The fragment 43 is not visible on the gel.

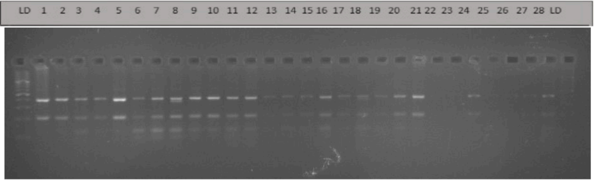

S2C Fig. OCTN1 rs3792876 polymorphism identified genetic variants of patients on RFLP analysis

Lane LD is representative of 100bp DNA ladder (Thermo-Scientific, USA); Major homozygous CC genotype (Lanes 1, 2, 3, 4, 5, 6, 7, 9, 10, 11, 12, 16, 17, 19, 20, 21, 24, 28); Heterozygous CT variant (Lanes 8, 18); The fragment 43 is not visible on the gel.
