# Supplementary material for: Lack of impact of OCTN1 gene polymorphisms on clinical outcomes of gabapentinoids in Pakistani patients with neuropathic pain
Source: PLoS One. 2022 May 13;17(5):e0266559. doi: 10.1371/journal.pone.0266559 (PMC9106170; doi:10.1371/journal.pone.0266559)
Supplement: Attachment — Submitted filename: Reponse to Reviewers.docx [file pone.0266559.s006.docx]

Cinzia Ciccacci
Academic Editor
PLOS ONE

Dear Editor,

We thank the editorial team and reviewers of PLOS ONE journal for their insightful and helpful comments on our manuscript titled "Lack of impact of OCTN1 gene polymorphisms on clinical outcomes of gabapentinoids in Pakistani patients with neuropathic pain" (PONE-D-21-32641). The suggestions of the academic editor and reviewer have been addressed and appropriate changes have been incorporated. The revisions are indicated in bold style in the manuscript and the references section. Following are the responses (written in Italic text) to each of the original comments made by the academic editor and reviewer.

**Response to Academic Editor’s Comments:**

**Journal Requirements:**

**When submitting your revision, we need you to address these additional requirements.**

1. **Please ensure that your manuscript meets PLOS ONE's style requirements, including those for file naming. The PLOS ONE style templates can be found at**
   [**https://journals.plos.org/plosone/s/file?id=wjVg/PLOSOne_formatting_sample_main_body.pdf**](https://journals.plos.org/plosone/s/file?id=wjVg/PLOSOne_formatting_sample_main_body.pdf) **and** 
   [**https://journals.plos.org/plosone/s/file?id=ba62/PLOSOne_formatting_sample_title_authors_affiliations.pdf**](https://journals.plos.org/plosone/s/file?id=ba62/PLOSOne_formatting_sample_title_authors_affiliations.pdf)

**Reply:** The manuscript has been revised according to PLOS ONE’s style requirements. The file names have been named according to the instructions.

**2. Thank you for stating the following financial disclosure:**

**The funders had no role in study design, data collection and analysis, decision to publish, or preparation of the manuscript.**

**At this time, please address the following queries:**

a) **Please clarify the sources of funding (financial or material support) for your study. List the grants or organizations that supported your study, including funding received from your institution.**
b) **State what role the funders took in the study. If the funders had no role in your study, please state: “The funders had no role in study design, data collection and analysis, decision to publish, or preparation of the manuscript.”**
c) **If any authors received a salary from any of your funders, please state which authors and which funders.**
d) **If you did not receive any funding for this study, please state: “The authors received no specific funding for this work.”**

**Please include your amended statements within your cover letter; we will change the online submission form on your behalf.**

**Reply:** *The statement about financial disclosure has been included in the cover letter of the manuscript.*

2. **We note that you have stated that you will provide repository information for your data at acceptance. Should your manuscript be accepted for publication, we will hold it until you provide the relevant accession numbers or DOIs necessary to access your data. If you wish to make changes to your Data Availability statement, please describe these changes in your cover letter and we will update your Data Availability statement to reflect the information you provide.**

**Reply:** *The Data Availability statement has been updated in the cover letter of the manuscript.*

**4. Please review your reference list to ensure that it is complete and correct. If you have cited papers that have been retracted, please include the rationale for doing so in the manuscript text, or remove these references and replace them with relevant current references. Any changes to the reference list should be mentioned in the rebuttal letter that accompanies your revised manuscript. If you need to cite a retracted article, indicate the article’s retracted status in the References list and also include a citation and full reference for the retraction notice.**

**Reply:** *Thank you for highlighting. The references list has been reviewed. We have not cited any retracted article. Some new references have been added in the discussion according to the suggestion of the reviewer and highlighted in the reference list of the manuscript.*

**Response to Reviewer’s Comments:**

**Reviewer #1: In the present paper, the authors investigated the possible influence of two OCTN1 SNPs on therapeutic efficacy and safety of gabapentinoids in neuropathic pain patients of the Pakistani population. They did not observe any significant association of genotypes and alleles of both SNPs on the clinical response to gabapentinoids. I think that the strategy followed is correct, the paper is well organized and tables are easily understood, although the authors find no association.**

**I have a few comments for the authors.**

**1. In the statistical analyzes, the heterozygous and homozygous for the variant allele subjects were merged into a single group, but it would be more complete to report the data of the single genotypic classes.**

**Reply:** *Thank you for your comment. The details of heterozygous and homozygous for the variant allele subjects have been reported separately in the results tables.*

2. **The authors should calculate the Hardy Weinberg equilibrium for the SNPs analyzed.**

**Reply:** *Thank you for the suggestion. We have provided the results of HWE analysis in relevant sections of the manuscript.*

**3. Authors should better explain a possible reasons for the effect of polymorphisms of OCTN1 on gabapentinoids efficacy.**

**Reply:** *Thank you for your valuable comment. The possible reasons for OCTN1 polymorphism on the efficacy of gabapentinoids have been added in the discussion segment of the manuscript.*

**4. The paragraph “PCR product electrophoresis” is superfluous, some of the information is already reported in materials and methods**

**Reply:** *Thank you for highlighting. The redundant lines have been removed from the results and appropriate information has been added in the materials and methods section.*

**5. Since the two studied SNPs are located in the same gene, the authors could analyze the frequencies of the haplotypes composed of these two genetic variants.**

**Reply:** *Thank you for your comment. The haplotype analysis of the two SNPs located in the same genetic region was conducted. There was no significant difference in the frequency of the haplotypes associated with the clinical outcomes so the results have not been included in the manuscript.*

**6. The response to drugs is a multifactorial trait. Other factors, genetic and otherwise, are known to be involved in the response to gabapentinoids? The authors could take them into consideration or at least mention them in the discussion.**

**Reply:** *We thank the reviewer for pointing this out. The suggestion has been addressed in the discussion part of the manuscript.*

**7. A revision of the English language is recommended, to correct mistakes and clarify some sentences.**

**Reply:** *The revision of English language has been done throughout the manuscript. Some sentences have been revised and shortened for clarity.*
